# Xenobiotic Compounds Degradation by Heterologous Expression of a *Trametes sanguineus* Laccase in *Trichoderma atroviride*

**DOI:** 10.1371/journal.pone.0147997

**Published:** 2016-02-05

**Authors:** Edgar Balcázar-López, Luz Helena Méndez-Lorenzo, Ramón Alberto Batista-García, Ulises Esquivel-Naranjo, Marcela Ayala, Vaidyanathan Vinoth Kumar, Olivier Savary, Hubert Cabana, Alfredo Herrera-Estrella, Jorge Luis Folch-Mallol

**Affiliations:** 1 Facultad de Ciencias, Universidad Autónoma del estado de Morelos, Av. Universidad 1001, Col. Chamilpa, Cuernavaca, Mor, México; 2 Centro de Investigación en Biotecnología, Universidad Autónoma del estado de Morelos, Av. Universidad 1001, Col. Chamilpa, Cuernavaca, Morelos, México; 3 Unidad de Microbiología Básica y Aplicada, Facultad de Ciencias Naturales, Universidad Autónoma de Querétaro, Anillo vial fray Junípero Serra km 8 s/n C.P. 76000, Santiago de Qro, México; 4 Instituto de Biotecnología, Universidad Nacional Autónoma de México, Av. Universidad 2001, Col. Chamilpa, Cuernavaca, Mor, México; 5 Department of Civil Engineering, Université de Sherbrooke, 2500 Boulevard de l’Université, Sherbrooke, Québec J1K 2R1, Canada; 6 Bioprocess laboratory, Department of Biotechnology, School of Bioengineering, SRM University, Kattankulathur 603203, Tamil Nadu, India; 7 Laboratorio Nacional de Genómica para la Biodiversidad, Cinvestav Campus Guanajuato, Km. 9.6 Libramiento Norte, Carretera Irapuato-León, 36821 Irapuato, México; Center for Nanosciences and Nanotechnology, MEXICO

## Abstract

Fungal laccases are enzymes that have been studied because of their ability to decolorize and detoxify effluents; they are also used in paper bleaching, synthesis of polymers, bioremediation, etc. In this work we were able to express a laccase from *Trametes (Pycnoporus) sanguineus* in the filamentous fungus *Trichoderma atroviride*. For this purpose, a transformation vector was designed to integrate the gene of interest in an intergenic *locus* near the *blu*17 terminator region. Although monosporic selection was still necessary, stable integration at the desired locus was achieved. The native signal peptide from *T*. *sanguineus* laccase was successful to secrete the recombinant protein into the culture medium. The purified, heterologously expressed laccase maintained similar properties to those observed in the native enzyme (Km and kcat and kcat/km values for ABTS, thermostability, substrate range, pH optimum, etc). To determine the bioremediation potential of this modified strain, the laccase-overexpressing *Trichoderma* strain was used to remove xenobiotic compounds. Phenolic compounds present in industrial wastewater and bisphenol A (an endocrine disruptor) from the culture medium were more efficiently removed by this modified strain than with the wild type. In addition, the heterologously expressed laccase was able to decolorize different dyes as well as remove benzo[α]pyrene and phenanthrene *in vitro*, showing its potential for xenobiotic compound degradation.

## Introduction

Laccases (EC 1.10.3.2) are multi-copper oxidases that have been extensively studied because of their great potential to oxidize phenols, polyphenols, aromatic amines and other non-phenolic compounds. Substrate oxidation can, in turn, generate free radicals able to diffuse away from the enzyme and non-enzymatically oxidize different compounds. These so-called mediators significantly contribute to the degradation of non-phenolic compounds present in lignin [1], [2]. Laccases are widely distributed in fungi, plants and bacteria, although the best studied come from white rot fungi since these organisms are able of mineralizing lignin and this feature has taken more relevance in the past few years for biofuel production from biomass [3]. Although peroxidases (also able of degrading a wide range of substrates) usually have a higher redox potential, laccases could be more suitable for some biotechnological purposes over other oxidizing enzymes (such as manganese-peroxidases, lignin peroxidases) since they use molecular oxygen as the electron acceptor, making the addition of hydrogen peroxide unnecessary. This also contributes to the stability of laccases, since peroxidases are eventually inactivated by H_2_O_2_ [4], [5]. Potential industrial application of laccases include decolorization and detoxification of effluents, textile-dye bleaching, polymer syntheses, lignocellulosic feedstock biorefinery applications and bioremediation [2].

In recent years, attention has been focused on several species of the genera *Pycnoporus* (recently renamed as members of the *Trametes* genus) since these white-rot fungi produce a number of biotechnological interesting secondary metabolites and enzymes (cinnabarin, chitinases, cellulases, amylases, etc.) and among them, laccases [6]. In our group, through the screening of a cDNA library, we cloned a laccase gene from a *Trametes sanguineus* strain (previously called *Pycnoporus sanguineus*) isolated from an oil-polluted tropical habitat [7]. The open reading frame deduced from the cDNA sequence of this laccase consists of 1557 bp coding for 519 amino acids and it shows conserved motifs common to all laccases, including the copper binding sites. A putative signal peptide was predicted from amino acid 2–19 with a probability of 0.997, being the most probable (0.959) cleavage site between amino acids 20 and 21. The purified enzyme from *T*. *sanguineus* showed activity towards 2,2’-Azino-bis(3-ethylbenzothiazoline-6-sulfonic acid) diammonium salt (ABTS), guaiacol, syringaldazine and *o*-dianisidine. It also showed high levels of activity in thermostability assays [7]. Heterologous protein expression in microbial hosts such as *Saccharomyces cerevisiae* is many times hindered probably due to differences in the glycosylation pattern of the enzyme [8]. *T*. *sanguineus* laccase glycosylation profile has been described in detail, consisting of high mannose structures, although the role of N-glycosylation only altered the enzyme stability, having little effect on other enzyme properties [9]. Numerous reports have proven that heterologous expression of laccases from fungal sources are difficult and low activities are obtained [10–13]. Several approaches have been used to overcome this limitation such as directed evolution of heterologously expressed laccases in yeasts [14], [15]. In one of this works, it was found that mutations in the signal peptide sequence were crucial for efficient expression [16].

*Trichoderma* species have been considered as good systems for heterologous expression of proteins because they can carry out eukaryotic posttranslational modifications and show good secretion capacities since they are efficient producers of extracellular enzymes that degrade a large variety of substrates and even xenobiotic compounds [17]. Some species of these fungi, *Trichoderma reesei*, for example, are already used for industrial production of cellulases and hemicellulases. *Trichoderma atroviride* has also been shown to be a good cellulase producer [18]. *Trichoderma* species genome analysis revealed that they contain several putative phenol-oxidase genes many of them being expressed intracellularly, depending on the species [19]. For certain *Trichoderma* strains it has been shown that extracellular laccase expression is nutrient medium dependent and up to 40mM CuSO_4_ must be added to induce the activity [20].

Many *Trichoderma* species have also been shown to be resistant to xenobiotic compounds [21], [22], so an efficient laccase-expressing strain could be desirable in order to bolster the applicability of this microorganism in lignocellulose utilization as well as bioremediation of contaminants.

Transformation techniques using protoplasts from germinating spores are the most widely used to introduce DNA into *Trichoderma* species. Integration takes place in random loci in the genome [23], which makes it necessary to analyze several transgenic lines. As most of the cells in *Ascomycetes* are at least dikaryotic [24], monosporic passes are needed to ensure a homogeneous population in which all of the nuclei are transformed.

In this work, we constructed a transformation vector that directs the stable integration of the gene of interest into a specific locus of the *T*. *atroviride* genome and used it to produce a strain that over expresses a laccase gene from *Trametes sanguineus*. We evaluated the potential of a representative transgenic strain and the heterologously expressed laccase for the transformation and removal of xenobiotic compounds from different sources such as bisphenol A (BPA), phenolic compounds in wastewater, benzo[*a*]pyrene and phenanthrene and industrial dyes.

## Results

### Analysis of vector pUE10 integration in *T*. *atroviride*

Vector pUE10 was constructed as described in Materials and Methods (S1 Fig). This vector directs the integration of the desired DNA fragment into a locus near the *blu*17 terminator intergenic region of *T*. *atroviride* (see discussion). Southern blot analysis showed that several empty pUE10 constructions and the laccase constructions tested were integrated downstream the *blu*17 terminator locus as predicted (data available upon request). Transformation with the pUE10 empty vector resulted in strains with a wild type phenotype as judged by the appearance of the mycelium, growth speed, mycoparasitic capabilities, conidiation, etc. (S2 Fig).

### Construction of *T*. *atroviride* strains expressing a *T*. *sanguineus* laccase

**Fig 1:**
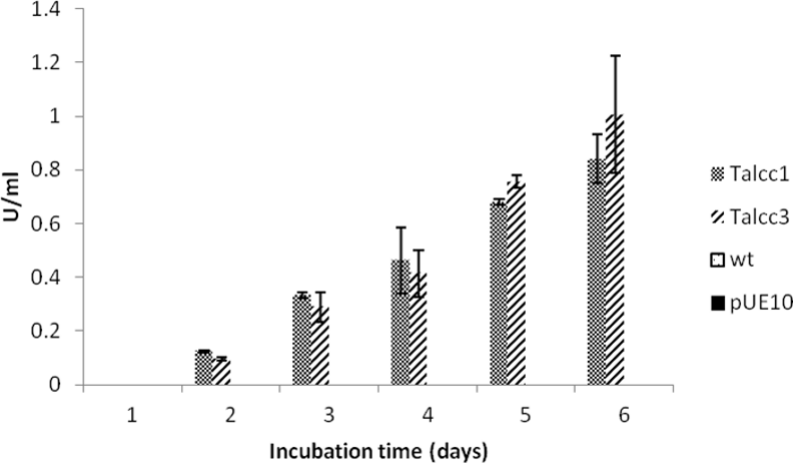
Laccase activity in the *T*. *atroviride* transformants. The wild type strain, a pUE10 transformant (empty vector) and *Ta*lcc*1* and *Ta*lcc3 transformants, strains were grown in Vogel’s minimal medium for six days and laccase activity was quantified. Bars show standard deviation.

The cDNA of the *T*. *sanguineus* laccase gene was cloned in vector pUE10, transformed into *T*. *atroviride* IMI206040 and after three monosporic cultures transformants were selected as described in materials and methods. Two transformant strains that showed an intense green color were selected for further study. These strains were grown in Vogel’s liquid medium and specific laccase activity was measured. The transformed strains studied showed similar activity (Fig 1), so *Ta*lcc3 was used for further analysis. The wild type *T*. *atroviride* strain and a strain transformed with pUE10 vector without an insert did not show any detectable laccase activity under any of the growth conditions used in this study, indicating that the measured activity corresponds to *T*. *sanguineus lcc*1.

### Characterization of the recombinant laccase

**Fig 2:**
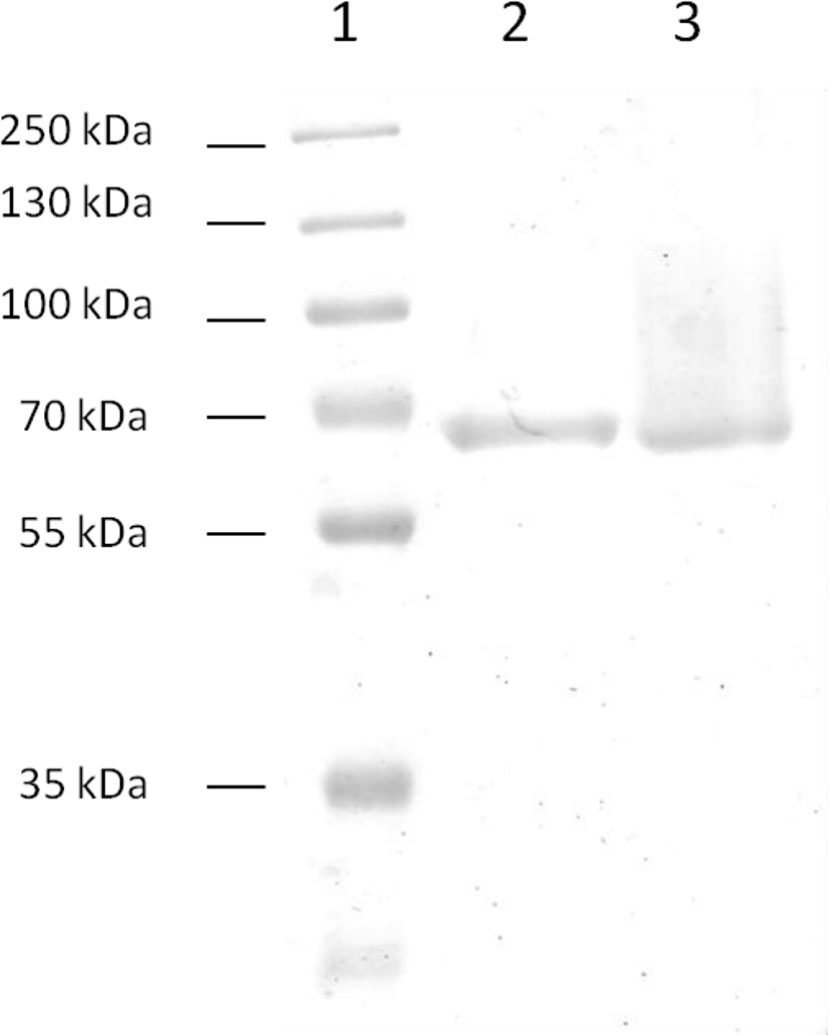
Purification of native and recombinant laccases. 1) Molecular weight markers (PageRuler^tm^ prestained protein ladder, Fermentas). 2) Native *T*. *sanguineus* laccase and 3) *Ta*Lcc3 laccase.

**Fig 3:**
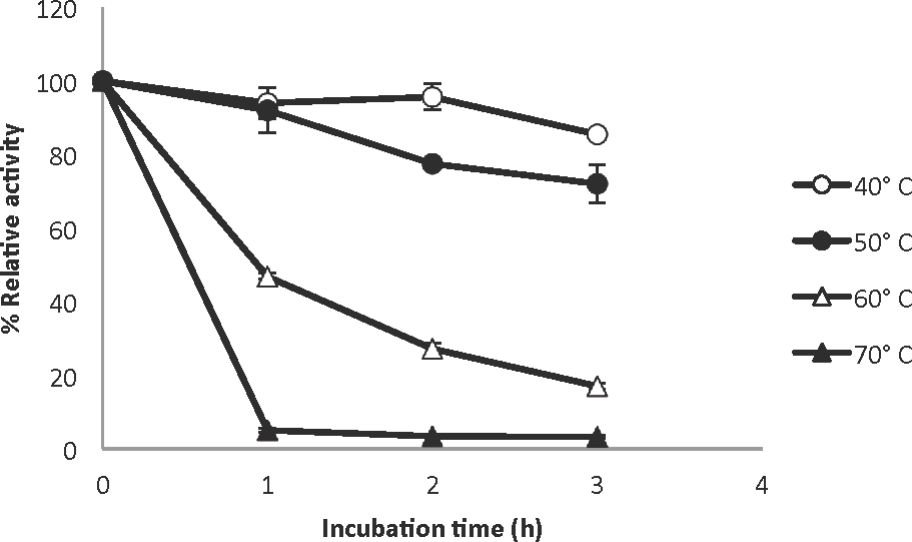
Thermal stability of the laccase produced by *Ta*Lcc3. Temperature effect on the laccase activity was determined by incubating the enzymatic extract at 40 (open circle), 50 (closed circle), 60 (open triangle) and 70°C (closed triangle) for 3 hours. Every hour a sample was collected to measure residual activity. Bars show standard deviation.

The native and recombinant laccases were purified as described in materials and methods (Fig 2). Kinetic parameters for ABTS were very similar for both enzymes (Table 1). Other properties of the heterologously expressed laccase were also similar to the native enzyme [7], although some differences were observed. The heterologously expressed protein seemed to be less thermostable than the native enzyme but still retained high levels of activity, with 92% and 46% of its original activity after incubation at 50°C and 60°C for 1 h, respectively; however, at 70°C activity was practically lost after one hour of incubation (Fig 3). Both enzymes showed a pH optimum of 4 (data not shown). Twenty four- hours incubation of the enzyme in buffers of different pH, ranging from 3 to 6, did not lead to activity loss (as measured at pH 4 after incubation; data not shown), showing that the recombinant enzyme is stable in this pH range. The recombinant laccase also showed activity towards syringaldazine, guaiacol, and 2,6-Dimethoxyphenol (2,6-DMP) (Table 2).

**Table 1:** Kinetics values for native and recombinant laccases, utilizing ABTS as a substrate.

Enzyme	substrate	Km (μM)	kcat (seg^-1^)	kcat/km
Native Lcc	ABTS	41.34 ± 1.47	76.70 ± 2.4	1.85
Recombinant Lcc	ABTS	48.10 ± 4.9	69.70 ± 3.47	1.44

**Table 2:** Recombinant laccase activity towards several substrates.

Substrate	Specific activity (U/mg)
ABTS	67.33 ± 3.2
Syringaldazine^a^	38.93 ± 5.46
2,6-DMP	82.43 ± 7.14
Guaiacol	15.39 ± 2.38

^a^ the reaction was measured in 100 mM succinate buffer pH 4.5.

### Xenobiotic degradation assays

**Fig 4:**
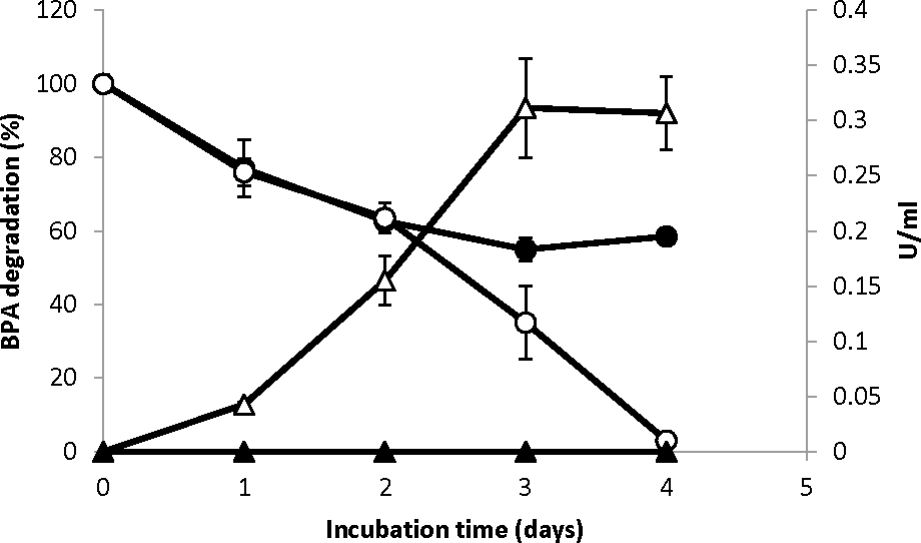
BPA removal from culture medium by *T*. *atroviride* strains. The wild type strain and *Ta*lcc3 were grown in Vogel´s minimal medium in the presence of 100 μM BPA for four days. Residual BPA in the supernatant of: the wild type strain (closed circles); the *Ta*lcc*3* strain (open circles). Laccase activity in the supernatant of: the wild type strain (closed triangles); the *Ta*lcc3 strain (open triangles). Bars show standard deviation.

The *Ta*lcc3 strain was tested for xenobiotic compounds degradation. Firstly, *Ta*lcc3 and the wild type strains were grown in the modified Vogel´s medium in the presence of 100 μM of bisphenol A, a powerful endocrine disruptant; higher concentrations (e.g. 1 mM) were lethal for both strains. During the first two days, both cultures showed a decrease in the concentration of soluble BPA; no laccase activity was detected for the wild type strain and already at day 2 laccase activity (0.15 U/mL) was detected in the *Ta*lcc3 culture (Fig 4). From day 2 to day 4, BPA was completely removed from the supernatant of the *Ta*lcc3 culture, while BPA concentration showed no further decrease in the wild type strain culture. Additional BPA removal from the *Ta*lcc3 culture was coincident with higher laccase activity in the supernatant (up to 0.35 U/mL) (Fig 4). Thus, it is apparent that the presence of extracellular laccase leads to a depletion of soluble BPA in the cultures. It is possible that the observed degradation of BPA in the wild type culture is due to adsorption to biomass. To test this idea, at the end of the culture and after removal of the supernatant, biomass of both strains was incubated in an ultrasonic bath for 30 min; BPA was detected exclusively in the supernatant of the wild type strain, whereas only traces of it were found for *Ta*lcc3 strain (data not shown).

Secondly, wastewater from a biofuel industry plant containing phenolic compounds (cresols, nitrophenols, chlorophenols, among others) was treated with both *Ta*lcc3 and wild type strains for 12 days at 28°C. *Ta*lcc3 removed 7% of the phenolic compounds in this water, while the wild type strain was not able to remove these compounds. However, when the wastewater was supplemented with 0.1 mM glucose the recombinant strain revealed a strong potential to eliminate phenolic compounds in the wastewater (35%), while the wild type strain only removed 11%. This results supports that the ≅25% of phenol degradation difference is due to the laccase heterologous expression of *T*. *sanguineus*. On the other hand, Phenanthrene and benzo[*α*]pyrene concentration was reduced by 97 and 99% respectively when incubated with the supernatants from the recombinant strain (57.5 U/L) for 24 h. Laccase activity and PAHs biodegradation were not observed when wild type supernatants were used (S3 Fig). The expressed laccase showed high potentialities to remove PAHs.

Finally, several dyes were also tested for transformation by the laccase produced by strain *Ta*lcc3 (1.7 U). Table 3 shows the results for the discoloration of Congo Red (CR), Bromophenol Blue (BPB), Coomasie Brilliant Blue (CBB) and Tripan Blue (TB). While no effect was noted on the CBB and TB decoloration, *Ta*lcc3 laccase decreased the BPB and CR concentration by 67.7 and 18.01% respectively (Table 1).

**Table 3:** Dye discoloration by 1.7 U of recombinant laccase after 12 hours of incubation.

Dye	Wavelenght (nm)	Discoloration (%)
BPB	592	67.73 ± 5.83
CR	504	18.01 ± 1.46
CBB	554	3.97 ± 1.52
TB	598	1.38 ± 0.86

## Discussion

Heterologous expression of proteins is an important alternative to overproduce an enzyme or to construct strains with novel capabilities. In some organisms, like bacteria and yeasts (however not in plants), directed integration of a DNA sequence in a specific locus in the genome is possible through mitotic homologous recombination. This feature is desirable because it facilitates the analysis of the transformant lines, since it reduces the variability in gene expression. If insertions of the desired DNA fragment occur in different loci of the genome by random recombination events in different lines, then chromatin structure, subtelomeric silencing, mutations or other phenomena can affect transgene expression, making it necessary to analyze a large number of independent transformants [12]. In this work we constructed a vector that allows directed integration of DNA in the genome of *T*. *atroviride* by adding an 804 bp sequence of a region downstream the *blu*17 terminator that enhances homologous recombination at this site. This proved to decrease the variability of laccase production among different transformant strains, since all the strains tested showed similar activities. It also shows that there is no impairment for expression at this locus. It is also important to note that transformants with the pUE10 empty vector showed a wild type phenotype indicating that integration of DNA in this locus did not cause any phenotypic alteration. Other groups have directed integration of DNA in *Trichoderma* species for different purposes [25], [26]. Strong expression of an endoglucanase was achieved in *Trichoderma reesei* when the endoglucanase gene was replaced at the *cbh*I locus, a strongly expressed cellulase in *T*. *reesei* [26]. In this work we achieved also a strong expression of the laccase using the *pki* promoter of *T*. *reesei*, indicating that this promoter works in *T*. *atroviride* and that this strain can express an enzyme that is usually difficult to express in heterologous systems (see below).

Laccases are enzymes that have been proven difficult to express in heterologous hosts, especially in yeasts, while the best results have been achieved using filamentous fungi such as *Aspergillus* and *Trichoderma* [27], [16], [10]. Moreover, we were able to successfully express in *T*. *atroviride* a *T*. *sanguineus* laccase that retains similar characteristics to the native enzyme. The native signal peptide sequence of *T*. *sanguineus*, although phylogenetically distant, allowed proper extracellular expression. It will be interesting to test if *T*. *sanguineus* laccase signal peptide is able to efficiently direct secretion of other proteins in *T*. *atroviride*. Other examples in which the native peptide signal was sufficient for proper secretion in heterologous systems have been described [13].

*Ta*lcc3 strain proved to be a good laccase producing strain so we decided to test its capabilities to degrade xenobiotic compounds. Fungal laccases are able to degrade endocrine disruptors such as bisphenol A [28], [29]. BPA is a compound present in polycarbonate plastics such as baby bottles, water bottles, food containers, etc. that has been found to leach and is now considered an environmental hazard [28]. Due to structural similarities, BPA mimics the activity of the hormone estradiol, leading to negative health effects. Endocrine-related effects have been detected in a wide range of concentrations, starting as low as 5 nM-5 μM [30]. The toxicity of BPA was also evident when cultures of both wild type and *Ta*lcc3 strains failed to grow in the presence of 1 mM BPA. Still, *Ta*lcc3 strain completely removed BPA (100 μM) while the wild type strain could only remove 62% and had no further capacity of removal after the first two days of culture. This initial removal could be due to a limited degradation by other pathways, such as cytochrome P450, present in *Trichoderma* species [31] and/or adsorption of BPA into the biomass. The idea of non-specific adsorption to the cell wall is supported by the fact that upon sonication, BPA was released from the wild type strain biomass; however, we did not observed a significant amount of BPA released from the *Ta*lcc3 strain biomass. Thus, complete BPA removal coincides with the increase of laccase activity in the *Ta*lcc3 culture, strongly suggesting that BPA is removed by the enzyme. Although phenol oxidases genes have been described in several *Trichoderma* species [19], it is worth to note that the wild type strain did not show induction of any extracellular laccase activity in any condition used in this study, even in the presence of CuSO_4_ or BPA. In fact, the products from the laccase-catalyzed reaction are mainly insoluble polymers, which precipitate from aqueous solution and thus may not be a readily available carbon source. The polymeric product shows no endocrine-related effects, as shown by others [32], thus laccase treatment effectively reduces the toxicity of BPA-contaminated water. Regarding other pollutants, the laccase produced by *Ta*lcc3 strain was also capable of decolorizing CR and BPB solutions *in vitro*. BPB has several phenol groups that could be electron donors for the enzyme-catalyzed reaction. On the other hand, azo compounds such as CR are known to be substrates of the enzyme [33]; it is interesting to note that TB, also an azo compound and even more polar than CR, was not substrate for the enzyme, probably due to steric hindrance of the azo moiety by the sulfonate group. It remains to be explored if laccase mediators in the culture medium could enhance the degradation capabilities of *Ta*lcc3 strain to extend the range of substrates, in order to oxidize challenging industrial dyes such as CBB. On other hand, the supernatant from recombinant laccase strain was able to remove phenanthrene and benzo[*α*]pyrene (97 and 99% respectively), these compounds are very toxic and the degradation by other white rot fungus has been studied [34]. In *Trametes versicolor* it was reported that the degradation of phenanthrene by a purified laccase from this fungus only took place when HBT or ABTS was added (40 and 30% respectively) [35]. In general the biotechnological potential of *Ta*lcc3 strain is very high and interesting, the capacity to grow in the presence of xenobiotic compounds and remove a high percentage of them, can be considered for using this strain directly for different processes for bioremediation.

## Conclusions

Directed integration of the *T*. *sanguineus* laccase gene has been proven to be an efficient and reliable strategy for construction of *Trichoderma atroviride* strains with enhanced bioremediation capabilities. It remains to test these strains in lignocellulosic material degradation as well as in plant colonization assays, which still would increase its biotechnological possibilities.

## Materials and Methods

### Strains, media and growth conditions

*Trichoderma atroviride* IMI206040 was used as the wild type strain in this work. *Trichoderma* strains (wild type or transformed) were propagated in PDA medium (DIFCO) at 28°C. When necessary, hygromycin was added to a concentration of 100 μg/mL. For liquid cultures, Vogel’s medium (with glucose 2% as a carbon source) [36] was inoculated with 1x10^7^ spores or 2 g of mycelium per 100 mL of medium and incubated at 28°C at 200 rpm. *Escherichia coli* DH5α was used routinely as a host strain for cloning vectors and plasmid propagation and purification. Standard molecular techniques were performed according to Sambrook et al., [37]. *E*. *coli* was grown in LB medium at 37°C and when required, carbenicillin (100 μg/mL) or chloramphenicol (34 μg/mL) were added. *T*. *sanguineus* CeIBMD001 was propagated in PDA medium or grown in liquid cultures as described previously to produce laccase [7].

### Construction of the integrative vector pUE10

A plasmid derived from pCB1004 [38] (pUE07) containing a chloramphenicol resistance gene and a hygromicin B resistance gene was used as the base for the construction of pUE10 [39]. pUE07 contains the *E*. *coli* f1 and colE1 origins, as well as a multiple cloning site and the *trp*C terminator of *Aspergillus nidulans*.

The *trpC* terminator sequence in pUE07 was replaced by a sequence containing the *blu*17 terminator and a region of 804 bp downstream of it (to direct integration by homologous recombination), by digestion with *Xho*I and *Kpn*I. The promoter of the *pki*1 gene from *T*. *reesei* was inserted in the *Sac*I and *Sac*II sites (S1 Fig). Two other versions of this vector with distinct inducible promoters (*pbr*1 from *T*. *atroviride* and *qa*2 from *Neurospora crassa*) were also constructed (data not shown).

### *T*. *sanguineus* laccase gene construction

The native laccase cDNA from *T*. *sanguineus* was amplified by PCR using as a template a former construction cloned in our laboratory [9] (GenBank accession number FJ513077). Primers TriLac1 (5’ *GCGGCCGC*ATGGGCTTCCGTCTTTCC 3’) and Lcc*Eco*RI (5’ *GAATTC*TTAATGGTCAGACTCCGGGA 3’) that contain the *Not*I and *Eco*RI sites respectively (in italics) were used to amplify a PCR product for further subcloning into pUE10. The PCR conditions were: 94°C 3’, 35 cycles of 94°C 45”, 54°C 1’ and 68°C 90” and one cycle of 10’ at 72°C. Several clones were sequenced to verify they were correct.

### Transformation and monosporic selection

Transformation was carried out according to Herrera-Estrella, et al., [23]. The transformed cells were spread on PDA plates containing hygromycin B in the presence of light until spores were obtained. These spores were diluted and re-plated for at least three times until stable lines were obtained. The transformant strains were named *Ta*lcc1, *Ta*lcc2, *Ta*lcc3, etc. (for ***T****richoderma* ***a****troviride* laccase (lcc)). All the tested strains showed equivalent laccase activities, so we chose *Ta*lcc3 for the rest of the experiments.

### Southern blot

Southern blot analysis of the transformant strains was performed as described in Sambrook et al., [37] using the RadPrime DNA labeling system (Invitrogen). DNA extraction was isolated following the procedure described by Raeder and Broda [40]. Genomic DNA was digested with *Nco*I and a 500 pb fragment kb containing the *blu*17 sequence was used as a probe.

### Laccase activity assays

Selection of laccase producing transformant strains was screened by growth in Vogel’s solid medium (the same as liquid but with 2% agar) plus 1mM ABTS (AMRESCO, Solon, OH, USA) at 28°C. Several strains that developed a green color were selected for further analysis (the wild type strain and the pUE10 transformant were used as negative controls and did not develop color at any stage of the experiment).

For laccase activity assays, strains were grown in Vogel’s liquid medium at 28°C and 200 rpm. When supplemented with 100 μM CuSO_4_ laccase activity increased, therefore this modified Vogel’s medium was used for the rest of the experiments (the wild type strain or pUE10 transformants did not show laccase activity even in this medium). For routine measurements, the initial ABTS oxidation rate (ε_436_ = 2.93 × 10^4^ M^−1^ cm^−1^) was measured in 100 mM sodium succinate buffer, pH 4 at room temperature using different volumes of culture supernatant (1–100 μL). Activity was also determined with guaiacol (ε_470_ = 2.6 × 10^4^ M^–1^ cm^–1^), syringaldazine (ε_530_ = 6.4 × 10^4^ M^–1^ cm^–1^) and 2,6-DMP (ε_468_ = 1.48 × 10^4^ M^-1^ cm^-1^). One unit of enzyme was defined as the amount of enzyme required to generate 1μmol of product per minute. Specific activities are reported as the number of μmol of product generated per milligram of protein per minute (μmol mg^−1^ min^−1^). All reactions were performed in triplicates. Protein quantification was performed using Lowry´s method [41].

### Purification of recombinant and native laccases

*Ta*lcc3 and *T*. *sanguineus* strains were grown in modified Vogel´s medium and Bran Flakes medium respectively. The supernatants were precipitated with 40% of acetone and 20% of ammonium sulfate. The precipitate was resuspended and concentrated with centricon membrane with a cut-off of 30 kDa. The concentrated sample was dialyzed overnight in 25 mM sodium acetate buffer, pH 5.5. This sample was loaded into a DEAE-sepharose column, equilibrated with 100 mM sodium acetate buffer, pH 5.5. All fractions with laccase activity were concentrated by ultrafiltration (centricon cut-off 30 kDa). Homogeneity of the enzymes was verified by SDS-PAGE. Laccase activity during the purification process was estimated by ABTS oxidation as described previously.

### Xenobiotic degradation assays

For bisphenol A (BPA) degradation assays, 2 g of mycelium of the wild type and the *Ta*lcc3 strains were inoculated in 100 mL modified Vogel’s medium (pH 6) supplemented with 100 μM BPA and incubated for 4 days at 28°C and 200 rpm. For laccase activity measurements, 100 μL were collected every 24 hours and the ABTS assay was performed as previously described. In parallel, a sample of the culture supernatant was periodically collected to quantitatively determine BPA concentration. BPA concentration was monitored using a HPLC-UV system at 225 nm with a C18 Phenomenex 100A column using a linear gradient of water:acetonitrile at a flow rate of 0.7 mL/min. The gradient program was 0–5 min acetonitrile 30%, 15–20 min acetonitrile 100%, and 21–26 min acetonitrile 30%.

For experiments dealing with the removal of phenolic compounds from a biorefinery process water, two plugs 7 mm diameter of the wild type (wt) and *Ta*lcc1 strains were inoculated in 50 mL of wastewater in 250 mL Erlenmeyer flasks. Two different systems were used and incubated for 12 days at 28°C and 150 rpm, wastewater supplemented with 0.1 mM of glucose and wastewater without glucose. The initial pH of the solution was adjusted to 7 by adding sulphuric acid. Phenolic compounds removal percentages for each strain were calculated after the phenol content estimation according to a previously reported method [42]. Abiotic controls were considered in the analysis. Three replicates of each culture were analyzed and three independent measurements for each case were made.

For tested of decoloration of industrial dyes (Congo red (Hycel, Mexico), bromophenol blue (Hycel, Mexico), Coomasie blue (Research Organics) and tripan blue (Sigma)) by the heterologously expressed laccase, *Ta*lcc3 strain was grown in 100 mL of modified Vogel´s medium for 4 days at 28°C and 150 rpm, the supernatant was concentrated with centricon membrane with a cut-off of 30 kDa. Dye solutions were prepared at 0.1% in citrate buffer pH 4, the reaction was started by adding 1.7 units of laccase in one mL final volume and absorbance was monitored at 507 nm, 592, 554 and 598 nm, respectively, after 12-hour incubation. To quantify the discoloration percentage the following formula was used: (initial absorbance- final absorbance) /initial absorbance X 100.

For removal of benzo[*α*]pyrene and phenanthrene, two plugs 7 mm diameter of each strain (recombinant and wild type) were inoculated in 50 mL of a 2% malt extract solution supplemented with 2% glucose contained in 250 mL Erlenmeyer flasks. The cultures were incubated for 4 days at 28°C and 150 rpm. The mycelium was removed by centrifugation at 12000 x g, and laccase activity was measured from the supernatants. Phenanthrene and benzo[α]pyrene were added into supernatants up to a 10 ppm (final concentration). Each treatment was incubated at 28°C and shaken at 150 rpm for 24 h. For the extraction of the selected PAHs, a liquid-liquid extractions were performed three times using 2 mL of the hexane and 2 mL of treated samples. The recovered hexane (final volume 6 mL) was concentrated 3 times using a vacuum rotatory evaporator. Gas chromatograph-mass spectrometer (GC-MS) was used to determine the PAHs concentration in each sample according with the previously reported methods [43], [44]. Abiotic controls were considered in the analysis. All experiments were made in triplicates.

## Supporting Information

S1 Fig pUE10 plasmid was constructed from pCB1004. First the *Aspergillus* terminator *trp*C was replaced by *blu*17 terminator and the *pki*1 promotor was inserted by restriction with *Sac*I and *Sac*II. This vector has resistance genes for hygromicin and chloramphenicol selection in *T*. *atroviride* and *E*. *coli* respectively. The *blu*17 terminator and the sequence downstream, allow the integration of this construction into a locus near the *blu*17 terminator intergenic region of *T*. *atroviride*.
(PDF)

S2 Fig Phenotypes of wild type and pUE10 strains in confrontations with *F*. *oxisporum*. (A) Confrontation between wild type (wt) strain and *F*. *oxisporum* (*Fo*). (B) Confrontation between pUE10 strain and *F*. *oxisporum*. The growth rate was 2.53 and 2.49 cm a day, respectively.
(PDF)

S3 Fig Chromatograms derived from the PAHs degradation. (A) *Trichoderma* WT-benzo[*α*] Pyrene. (B) *Trichoderma* WT-phenanthrene. (C) *Trichoderma* Lac- benzo[*a*] Pyrene. (D) *Trichoderma* Lac-phenanthrene.
(PDF)
