# Supplementary figures and images for: Xenobiotic Compounds Degradation by Heterologous Expression of a Trametes sanguineus Laccase in Trichoderma atroviride
Source: PLoS One. 2016 Feb 5;11(2):e0147997. doi: 10.1371/journal.pone.0147997 (PMC4743974; doi:10.1371/journal.pone.0147997)

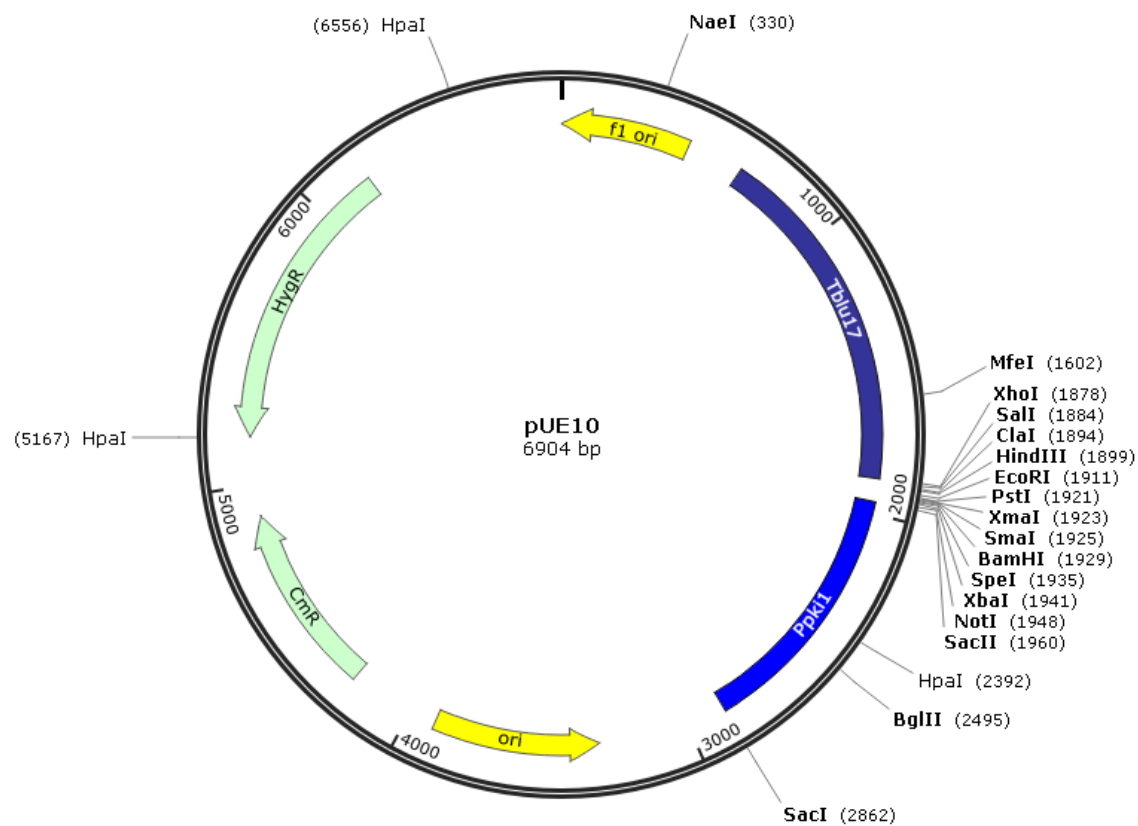

Supplement: S1 Fig — First the Aspergillus terminator trpC was replaced by blu17 terminator and the pki1 promotor was inserted by restriction with SacI and SacII. This vector has resistance genes for hygromicin and chloramphenicol selection in T. atroviride and E. coli respectively. The blu17 terminator and the sequence downstream, allow the integration of this construction into a locus near the blu17 terminator intergenic region of T. atroviride. (PDF) [file pone.0147997.s001.pdf]

A

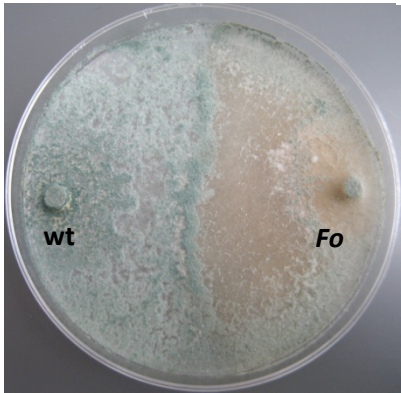

B

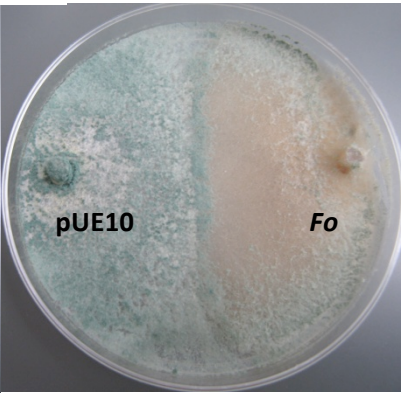

Supplement: S2 Fig — (A) Confrontation between wild type (wt) strain and F. oxisporum (Fo). (B) Confrontation between pUE10 strain and F. oxisporum. The growth rate was 2.53 and 2.49 cm a day, respectively. (PDF) [file pone.0147997.s002.pdf]

(A)  
Abundance

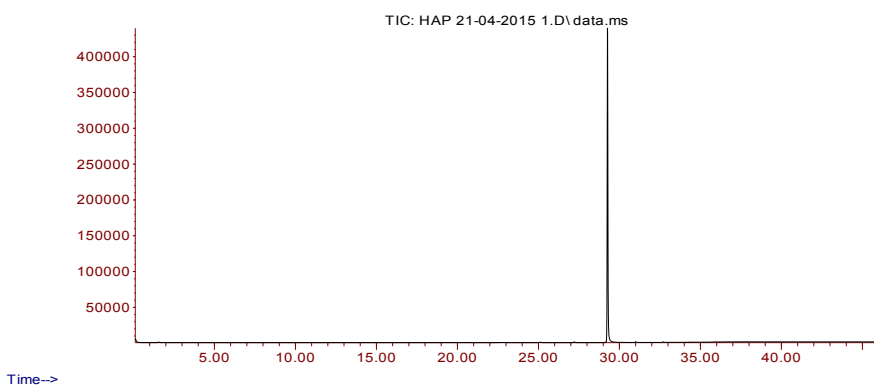

(B)  
Abundance

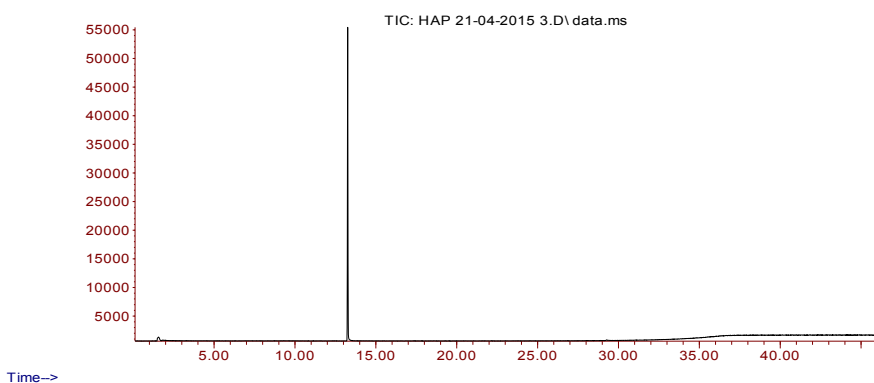

(C)  
Abundance

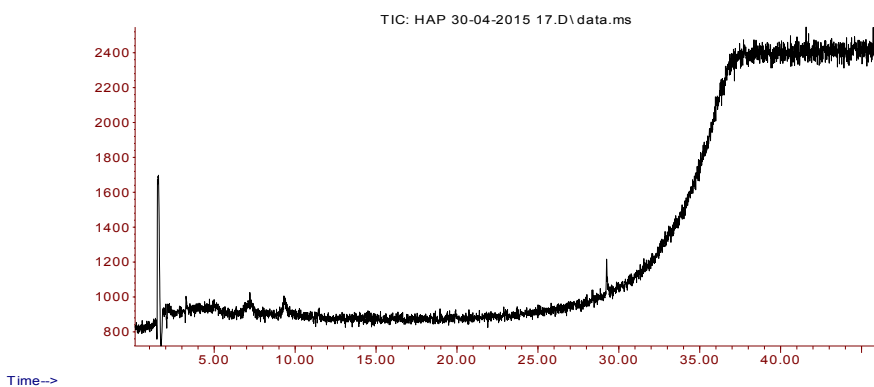

(D)

Abundance

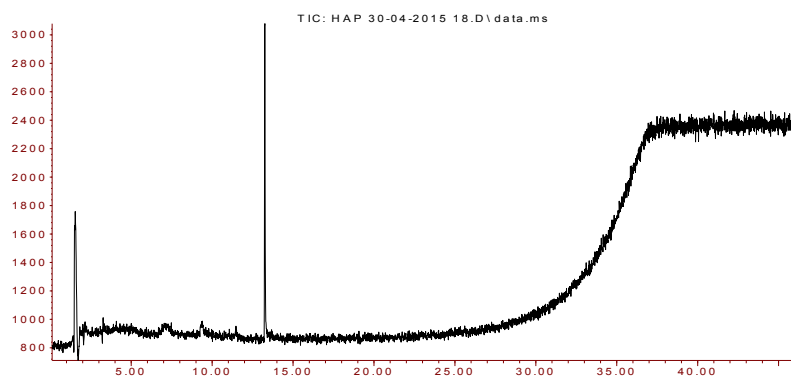

Supplement: S3 Fig — (A) Trichoderma WT-benzo[α] Pyrene. (B) Trichoderma WT-phenanthrene. (C) Trichoderma Lac- benzo[a] Pyrene. (D) Trichoderma Lac-phenanthrene. (PDF) [file pone.0147997.s003.pdf]
